# Tuning the peak position of subwavelength silica nanosphere broadband antireflection coatings

**DOI:** 10.1186/1556-276X-9-361

**Published:** 2014-07-19

**Authors:** Fei Tao, Pritesh Hiralal, Lianbing Ren, Yong Wang, Qing Dai, Gehan AJ Amaratunga, Hang Zhou

**Affiliations:** 1 School of Electronic and Computer Engineering, Peking University Shenzhen Graduate School, 2199 Lishui Road, Shenzhen, Guangdong 518055, China; 2 Electrical Engineering Division, Department of Engineering, University of Cambridge, 9 JJ Thomson Avenue, Cambridge CB3 0FA, UK; 3 Guangdong Key Lab of Nano-Micro Materials Research, School of Chemical Biology & Biotechnology, Peking University Shenzhen Graduate School, 2199 Lishui Road, Shenzhen, Guangdong 518055, China; 4 National Center for Nanoscience and Technology, Chinese Academy of Sciences, No.11 ZhongGuanCun BeiYiTiao, Beijing 100190, China

**Keywords:** Peak tunable, Antireflection, Light trapping, Spheres, Langmuir-Blodgett, Solar cells

## Abstract

Subwavelength nanostructures are considered as promising building blocks for antireflection and light trapping applications. In this study, we demonstrate excellent broadband antireflection effect from thin films of monolayer silica nanospheres with a diameter of 100 nm prepared by Langmuir-Blodgett method on glass substrates. With a single layer of compact silica nanosphere thin film coated on both sides of a glass, we achieved maximum transmittance of 99% at 560 nm. Furthermore, the optical transmission peak of the nanosphere thin films can be tuned over the UV-visible range by changing processing parameters during Langmuir-Blodgett deposition. The tunable optical transmission peaks of the Langmuir-Blodgett films were correlated with deposition parameters such as surface pressure, surfactant concentration, ageing of suspensions and annealing effect. Such peak-tunable broadband antireflection coating has wide applications in diversified industries such as solar cells, windows, displays and lenses.

## Background

Antireflection coatings (ARCs) have important roles in a wide range of industrial applications such as solar cells, buildings, smartphone displays and camera lenses. Current ARC technology, which based on destructive interference mechanism, usually requires costly vacuum deposition techniques such as sputtering or chemical vapour deposition. Recently, subwavelength nanostructures, such as nanowires, nanospheres and nanorods, resulting in a graded refractive index, emerged as ideal optical structures for ARC application. Among these, silica spheres with controllable diameter ranging from 50 nm to 2 µm prepared by Stober method have been the most studied [1-5]. Silica nanospheres could be used as etching mask [6,7] to create graded refractive index nanowire/nanodome structures, or nanospheres themselves could be used as antireflection coatings directly [8,9]. Optimized refractive index of single AR film was given by the equation $n_{\text{c}}=\sqrt{n_{\text{a}}n_{\text{s}}}$, where *n*_a_ and *n*_s_ are the refractive index of the air and the substrate, respectively. Commercial borosilicate glass substrate typically has a refractive index approximately 1.51, which means that a material with a refractive index approximately 1.23 is required in order to get the AR effect between air and glass. Given the fact that no material with such low refractive index has been discovered, most researchers have adopted mesoporous or hollow silica spheres to get the desired low refractive index [4,10,11]. Few attention were paid to the solid silica nanospheres. It is questionable whether thin films composing solid silica spheres, in particular for monolayer of silica nanospheres, could lead to remarkable AR effects.

Several methods have been employed to deposit nanosphere films on various substrates, including continuous assembly [12], convective assembly [5,13], layer by layer method (LbL) [3,4], printing [14] and Langmuir-Blodgett method [15,16]. Among them, Langmuir-Blodgett (LB) method is the most convenient and effective approach for controllable deposition of ordered nanospheres. It has been commonly used to make two-dimensional (2D) and three-dimensional (3D) photonic crystal structures. Bardosova et al. reviewed the monolayer and multilayer deposition of silica spheres by LB method [17]. Although the mechanism behind LB deposition and self-ordering thin film has been discussed for a number of years [17-19], it is unclear how the LB deposition parameters could affect the AR performance of deposited nanosphere thin films.

In this study, we focused on 2D solid silica sphere film made by LB technique and its superior antireflection effect. A parametric study of deposition conditions is conducted and correlated to the resulting film morphology and optical properties. We demonstrated that the thin films of single-layer solid silica nanospheres with a diameter of approximately 100 nm could offer comparable AR effect with respect to the mesoporous counterparts. Furthermore, the transmission peak of the nanosphere silica AR coating can be controlled by varying the LB deposition parameters. To our best knowledge, no such peak-tunable property has been reported before, although spectral shift due to the thickness of mesoporous silica spheres’ thin film has been observed in previous works [4,5,9,10]. The deposition parameters which determine the peak transmission wavelength are extracted. Three variables, namely deposition pressure, surfactant concentration and solution ageing, were found to strongly correlate with the maximum transmission position. Film density and aggregations of nanospheres resulting from the above variables are considered as principal determining factor behind this shift. The ability of achieving broadband transmission and simultaneously being able to determine the position of maximum transmission (>99%) opens the possibility of many application-specific solutions. For photovoltaics, for instance, it is possible to match the absorption peak of absorber materials by tuning the transmission peak of glass. For displays, it can reduce reflection and glare, while transmitting more of the display light, thereby requiring lower intensity light and reducing energy consumption.

## Methods

All chemicals were used as received, without any further purification. Aqueous suspension of silica spheres (50 mg/ml, polydispersity index <0.2, diameter 100 nm) were purchased from Kisker Biotech GmbH & Co, Steinfurt, Germany. The silica sphere suspension was diluted down to 10 mg/ml with pure ethanol (ACS reagent, ≥99.5%, absolute, Sigma-Aldrich, St. Louis, MO, USA) and then mixed with hexadecyltrimethylammonium bromide (CTAB; ≥98%, Sigma-Aldrich). CTAB was used to change the hydrophilic/hydrophobic nature of the silica spheres. The final diluted suspension with CTAB was ultrasonicated for 30 min each time before deposition.

Microscope glass slides (Agar Scientific, Essex, UK, 76 mm × 26 mm) were cleaned in acetone, IPA and DI water subsequently in an ultrasonic bath for 10 min at each step. After cleaning, glass slides were treated with oxygen plasma (Philips RIE, New York, USA). Both sides of the slides were treated by 100-W O _2_ plasma for 5 min at a pressure of 150 mbar. Monolayer of silica nanospheres were deposited onto plain glass slides using a Langmuir-Blodgett trough (NIMA Technology model 612D, Coventry, UK). The deposition process and mechanism has been discussed by many previous reports [17-19]. After deposition, the samples were washed in ethanol for 10 min twice in order to remove the residual CTAB. Finally, the samples were blow-dried with nitrogen gas.

Optical transmission measurements were made using a Thermoelectron Corporation UV/VIS Spectrometer UV2 double beam spectrophotometer (Waltham, MA, USA). All transmission measurements here shown are with respect to air reference. Spatial arrangement of the silica spheres was characterized by scanning electron microscope (SEM; Zeiss EVO 50, Oberkochen, Germany). Finite-difference time-domain (FDTD) simulation (FDTD solutions, Lumerical Solutions, Inc., Vancouver, Canada) was used to verify the experimental results. The simulation software is a 3D computer-based Maxwell solver. Transmittance spectra of SiO _2_ nanosphere array with cubic arrangement on single side and double sides of glass were simulated. Details of simulation parameters are shown in Additional files 1, 2, 3 and 4.

## Results and discussion

**Figure 1:**
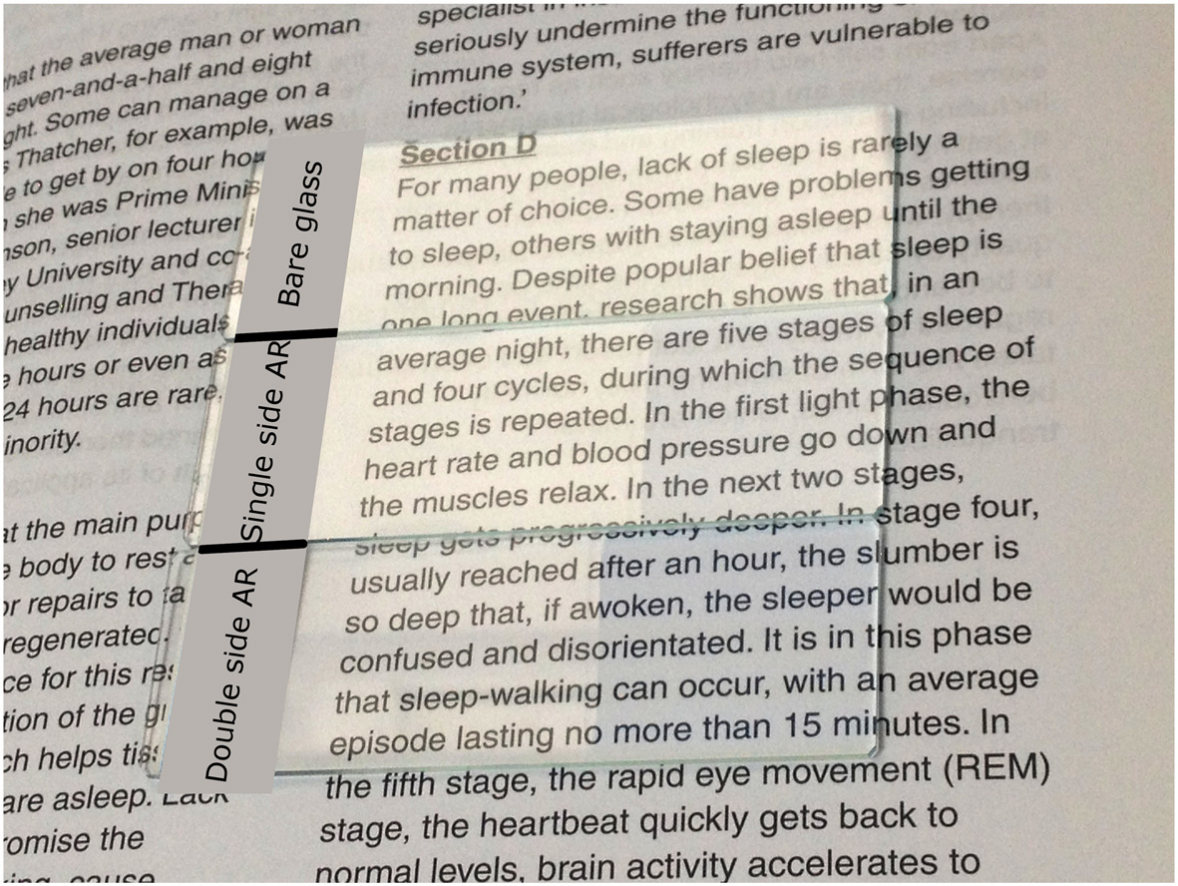
Digital photographs of bare glass, single-side AR and double-side AR on a piece of paper with texts.

AR film was deposited at a pressure of 20.0 mN/m using fresh prepared 1.0 mM CTAB suspension. Clear visual observation of the light-transmitting properties of the nanosphere coating can be seen in the digital photographs in Figure 1. In this figure, three samples were placed over a piece of white paper with black texts. On top is the bare glass sample. In the middle, there is a sample with its right part coated with single-side AR coating. The bottom sample is a sample with its right part coated with double-side AR coating. The figure visually demonstrate that the transmittance of the coated glass is higher than the bare glass and is highest when the glass is coated on both sides (double AR). Glare is obvious on all bare glass parts on the samples, while it was reduced on single AR and double AR samples. Comparing single AR and double AR, the AR effect was more pronounced in the double AR sample, as a result of the improvement of both abrupt interfaces of glass by the nanospheres. In addition, it can be also demonstrated that reflection was significantly reduced by coating double-side nanospheres (see Additional file 1: Figure S1).

**Figure 2:**
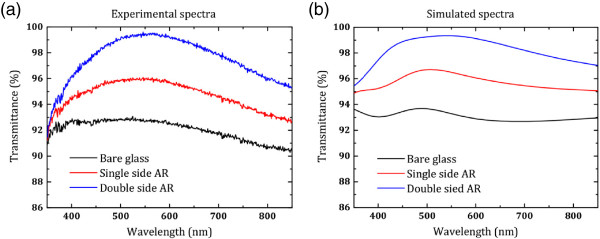
**Transmission spectra of bare glass, single AR and double AR. (a) ** Experimental results. **(b)** Simulated results.

The AR effects of single-side and double-side silica nanosphere coating were further confirmed by measuring transmission spectra of the samples. Transmission spectra of bare glass, single AR and double AR are shown in Figure 2a. Transmittance of bare glass was around 92% over the whole visible spectrum. Single-side AR-coated glass had higher transmittance than that of the bare glass with a peak value of approximately 95% at 560 nm. The double-side AR-coated glass had the highest transmittance, with a peak of approximately 99% at 560 nm. These experimental results are consistent with previous reports [4,9]. The 560-nm transmission peak on the AR-coated glasses is the main cause of the disappearance of the reflected images as the maximum spectral sensitivity of human eyes is at approximately 555 nm. FDTD simulation was used to verify the AR effects of silica nanosphere coating. Simulated transmission spectra are shown in Figure 2b. The general trend of the simulated curve matches our experimental data, though there are some mismatch probably due to the material index used in the model which are not identical to the real situation. Both experiments and simulation confirmed that thin films composing subwavelength silica nanospheres have superior antireflection effect on the interface between air and planar glass and that each optically abrupt interface should be taken into account in order to obtain the best antireflection performance.

To further control the transmission peak position of the glass with AR coatings, we studied several key LB deposition parameters, including deposition pressure, concentration of CTAB, compression-relaxation cycles and dipper speed. The annealing effect on the thin films and the effect of ageing the sphere-CTAB suspension were also studied.

**Figure 3:**
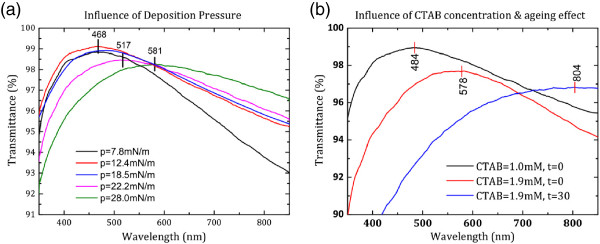
**Transmission spectra. (a)** AR films deposited at different pressures. **(b)** AR films deposited from fresh suspension with 1.0 mM, fresh suspension with 1.9 mM CTAB concentration and ageing suspension with 1.9 mM CTAB.

The influence of surface pressure during deposition on the transmission of the samples was investigated. Surface pressure of the mixed liquid is determined by the interaction between nanospheres. Surface pressure *π*_*A*_ is given by equation *π*_*A *_= *γ*_0 _- *γ*, where *γ*_0_ is equal to the surface tension of the water and *γ* is the surface tension of water with monolayer nanospheres. When the nanospheres are sufficiently far from each other, the resulting surface pressure is therefore very low, with measured pressure values similar to the pressure of pure water (*γ *= 71.97 mN/m at 25°C). When the average distance between spheres was reduced due to compression, surface pressure increased rapidly as a result of the strong interaction between spheres, i.e. adding a monolayer to the surface reduces the surface tension (*γ *< *γ*_0_). Further compression would cause monolayer collapse, forming nanosphere aggregations. Surface pressure just before the collapse of monolayer is known as collapse pressure. Collapse pressure of silica nanospheres in this experiment was 19 mN/m. Deposition pressures both under and above collapse pressure were studied. Figure 3a shows the transmission spectra of glass coated with AR films deposited at five different pressures. The pressures of 22.2 and 28 mN/m are both higher than collapse pressure, whereas all other three pressures are lower than collapse pressure. Three distinct peaks can be seen in the figure (468, 517 and 581 nm). Transmission peak was the same for samples deposited with pressures below collapse pressure (i.e. *p* = 7.8, 12.4 and 18.5 mN/m), while for samples deposited above this value (*p* = 22.2 and 28.0 mN/m), a shift in peak transmission position, which is a function of deposition pressure, was shown. For samples deposited below collapse pressure, the same spectral peak indicates that they are all thin films with monolayer nanospheres. The sample deposited at 7.8 mN/m had lower transmittance than the other two samples in long wavelength range, which may be due to the lower coverage of nanospheres on plain glass. We suspect that nanosphere aggregations formed when pressure went higher than collapse pressure, which caused the shift of transmission peak. Thus, samples deposited at *p*= 22.2 and 28.0 mN/m were nanospheres with different aggregation degrees rather than monolayer film of nanospheres.

**Figure 4:**
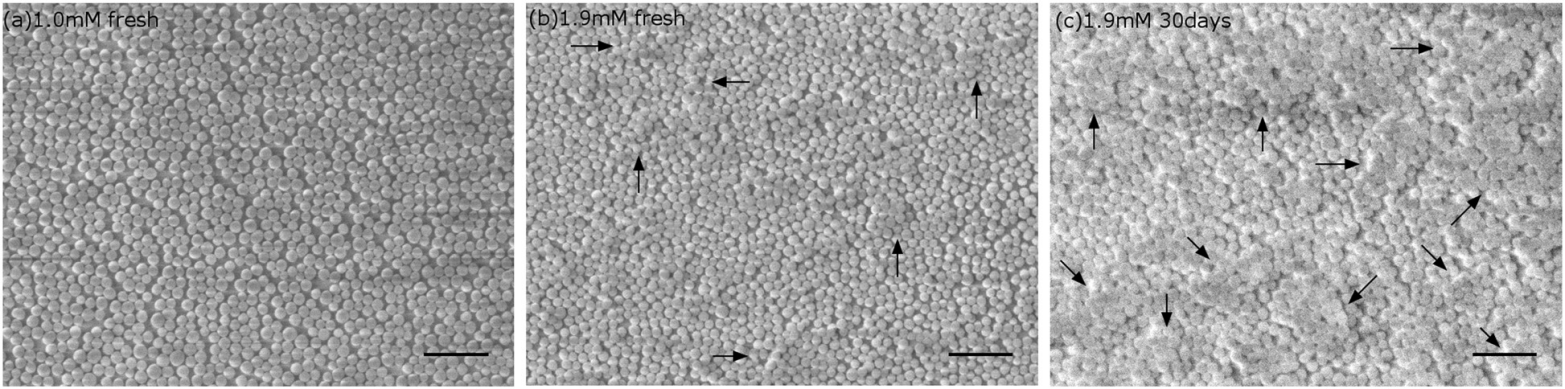
**SEM images. (a) ***C*_CTAB _= 1.0 mM fresh suspension. **(b) ***C*_CTAB _= 1.9 mM fresh suspension. **(c) ***C*_CTAB _= 1.9 mM ageing suspension. Aggregations were indicated by black arrows. Scale bar = 500 nm.

Concentration of surfactant, CTAB in this study, is another important parameter in the deposition process. The influence of concentration of surfactant on the optical transmission of the resulting film was studied. Bardosova et al. [20] reported on the deposition of colloidal crystals of silica particles by the LB method without using surfactant, providing the diameter lies in the range 180 to 360 nm. We found that, on the one hand, without surfactant, deposition of 100-nm nanospheres on glass slides was difficult to achieve; on the other hand, high concentration of CTAB cause aggregations of nanospheres during deposition. Suspensions with CTAB concentrations of 1.0 and 1.9 mM were used to investigate its influence on AR performance. The effect of solution ageing was investigated by preparing a suspension of 1.9 mM CTAB and using it to deposit at *t* = 0 and 30 days. Transmission spectra are shown in Figure 3a in which a peak shift can be found between the three spectra. The spectral peak shifted from 450 to 550 nm by increasing CTAB concentration from 1.0 to 1.9 mM. Ageing suspension was also found to cause the peak shifts. Given the same CTAB concentration of 1.9 mM, AR film deposited from fresh suspension and from ageing suspension (30 days old) showed different transmission peaks. The peak shifted from 578 to 804 nm as shown in Figure 3b. We suspect that the solution aggregates over time, which leads to aggregations in the thin films and the peak shifts. This assumption was supported by our SEM image analysis. SEM images of the three samples were given in Figure 4a,b,c. Image processing software (ImageJ) was used to estimate the coverage of the nanospheres. The area covered by the nanospheres was found to be approximately 78.90%. Assuming that nanospheres are monodispersed with a diameter of 100 nm, we are able to calculate the volume ratio occupied by nanospheres, which is 52.61%. A simple weighted model was used to calculate the equivalent refractive index of the monolayer silica spheres since the sphere diameter and the film thickness were both 100 nm which is small enough compared to the wavelength of visible light. The equation was given by $\varepsilon_{\text{eq}}=\varepsilon_{\text{air}}V_{\text{air}}+\varepsilon_{{\text{SiO}}_{2}}+V_{{\text{SiO}}_{2}}$, where *ε *_air_ and $\varepsilon_{{\text{SiO}}_{2}}$ are the dielectric constant of air and silica, and *V*_air_ and $V_{{\text{SiO}}_{2}}$ are their volume ratio. Then, the equivalent refractive index *n*_eq_ was estimated by the equation $n_{\text{eq}}=\sqrt{\varepsilon_{\text{eq}}}$. Given the refractive index of silica nanosphere is 1.45, the equivalent refractive index was calculated at *n*_eq _≃ 1.257. Refractive index of the glass slide is 1.5171, according to the specification from the seller. In previous theory, optimized refractive index of a single-layer AR film was estimated by $n_{\text{AR}}=\sqrt{n_{\text{air}}n_{\text{glass}}}$ if it is sandwiched between air and glass. Therefore, the optimized refractive index of AR material for this kind of glass slide is about 1.232, which is very close to the equivalent reflective index of our AR film. This explains the reason why sample using fresh suspension with 1.0 mM CTAB (black line) had the best integrated AR performance. It is clearly shown from Figure 4a that this sample is a monolayer of silica spheres without visible aggregations. However, for concentration of 1.9 mM, a few small aggregations can be seen in the film as indicated by the black arrows. The comparison between fresh suspension and ageing suspension gave similar aggregation evidence. Figure 4c shows that the aggregation degree was higher, and the aggregation size was larger compared to samples deposited from fresh suspension. The presence of aggregations will increase the volume ratio of silica nanospheres since aggregations are densely packed with volume ratio up to 74% (pack density of close-packing), which is much higher than 52.61% for our monolayer sample. Thus, aggregations consequently increase the equivalent refractive index of the AR film to *n*_eq _> 1.257, which will be even larger than the optimized value 1.232 and undermine the integrated AR effect.

It is noted that in our experiments the arrangement was not perfect close-packed but amorphous alike. This is due to the high polydispersity (<20%) of the silica nanospheres. Jiang et al. found that in their work when samples with slightly broader size distributions (>8%) are deposited, grain boundaries in the plane parallel to the substrate are observed [21]. It is believed that the monodispersity of the colloids, rather than the deposition process itself, is responsible for their long-range ordering. Agod et al. investigated the effect of polydispersity on the anisotropy and the fluctuation of the surface pressure tensor in Langmuir films during uniaxial compression [22]. They found that domain-structured films can form only below 7% to 8% polydispersity; beyond this limit, the particulate films have rather amorphous structure. As a result, we conclude that the non-perfect close-packed arrangement was a result of the high polydispersity index of the silica spheres. Nevertheless, the subwavelength structure showed excellent antireflection performance.

In addition, isotherm of the fresh and ageing suspension was also found to be different. Isotherm of ageing suspension gave much higher collapse pressure, which may indicate that the surface tension of water with monolayer nanospheres *γ* was further decreased by aggregated CTAB molecules and nanospheres. These results show that the shift of the transmission peak is strongly influenced by the aggregations introduced by CTAB. This is in agreement to the report by Yang et al. [23] who found that the concentration of CTAB in gold colloids is critical for self-assembling linear chain-like aggregates with different interconnecting particle number and network-like aggregates. In light of this phenomenon, we believe it is possible to control the transmission peak position via controlling the aggregation rate and size of the nanospheres. Another three variables including compression-relaxation cycles, dipper speed and annealing effect were found to have a weak correlation with peak position. Although increasing the number of compression-relaxation cycles of the spheres in water is known to produce a more compact film [24], transmission spectra of samples deposited with or without using compression-relaxation cycles were hard to distinguish (see Additional file 3). Situations of the other two parameters are similar. Given the fact that these three parameters have no effect on the formation of aggregations, it is consistent with our previous analysis that aggregation rate and size are the main factors determining the peak position.

According to the analysis above, deposition pressure, surfactant concentration and solution ageing have a strong correlation with the position of peak transmittance of the resulting coating. By varying these parameters, it was possible to tune the transmission peak position from 468 nm to beyond 800 nm, covering most of the visible spectrum.

**Figure 5:**
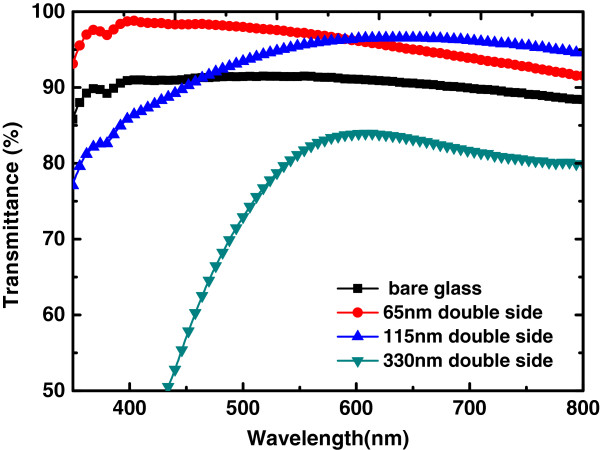
Effects on the radius of the nanospheres on the transmission spectra.

The radius of the nanosphere also have pronounced effect on the transmission peaks of the AR layer. When the radius of the spheres are much smaller (<300 nm) than the wavelength of light under concern, the incoming photons will see the surface as an effective medium. However, when the radius of the sphere becomes comparable to the visible wavelength, scattering of light will become significant. Effects on the radius of the nanospheres on the transmission spectra were measured and shown in Figure 5. The small-diameter (65 and 115 nm) silica nanospheres shows excellent AR performance over the visible range, whereas the silica nanospheres with 330-nm diameter lower the overall transmission spectra compared to a plain glass slide. Reports on light cavity enhancement effect are mainly for spheres with diameter at the wavelength scale, such as 600 nm [25,26], where whispering gallery modes in the spheres can be coupled into guided modes in the photoabsorbing layer. Here, in the absence of photoabsorbing layer, the light in the cavities will be re-emitted and being seen as scattering photons. Therefore, the effect of radius of the spheres will change the transmission spectrum of the AR layer on glass substrate, scattering lights with comparable wavelength.

## Conclusions

In summary, antireflection (AR) films were deposited on glass substrates using 100-nm silica nanospheres by Langmuir-Blodgett method. Double-side subwavelength nanosphere films showed excellent broadband AR effect which improved sample transmittance to higher than 95% in the whole visible spectrum, with transmittance peak higher than 99%. Furthermore, the spectral position of transmission peak can be tuned by controlling three key deposition parameters (deposition pressure, surfactant concentration, ageing of suspension). It is possible to tune the transmission spectral peak widely across the whole visible spectrum. Aggregations of nanospheres were ascribed to be the cause for this peak-tunable property according to our investigation. Transmission peak shifts to longer wavelength as the size and rate of aggregation increases. We believe that such peak-tunable broadband antireflection effect has huge potential for many application areas, such as solar cells, LED and displays.

## Competing interests

The authors declare that they have no competing interests.

## Authors’ contributions

FT deposited the samples, performed the spectral measurements and wrote the article. PH performed the SEM characterization. LR and YW provided silica spheres for testing. QD provide Langmuir-Blodgett trough for film deposition. PH, GAJA and HZ participated in the study guidance and paper revision. All authors read and approved the final manuscript.

## Supplementary Material

Additional file 1 **Digital photographs of reflected images.** In this figure, a mobile phone, which laid at the bottom, was used as the dark background. Glass samples with monolayer silica nanosphere coatings were laid on top of the mobile phone. A second smartphone with its built-in camera was used to take the photos. Therefore, the bare glass with high reflection would show the image of the photo-shooting smartphone camera. In Additional file 1: Figure S1(a), the left part of the glass sample was coated with single-side nanospheres, whereas in Additional file 1: Figure S1(b), both sides of the left part of the glass samples were coated with nanospheres. The right part of the glass in both Additional file 1: Figure S1(a) and S1(b) were left untreated for comparison, where reflecting image of the smartphone camera were clearly observed. The figure shows partially coated glass slides placed over mobile phone. Additional file 1: Figure S1(a) shows a glass slide with a silica nanosphere AR coating on a single side (single AR), while the glass slide on Additional file 1: Figure S1(b) is coated on both sides (double AR). Both samples are partially covered with the remaining glass left bare to observe the difference. These figures visually demonstrate two striking effects. Firstly, the transmittance of the coated glass is higher than the bare glass and is highest when the glass is coated on both sides (double AR). Secondly, the reflectivity, observed in the pictures as the reflection of the photo-taking camera, is reduced on the coated samples. No reflected image could be found on the double AR part of glass region in Additional file 1: Figure S1(b). Comparing Additional file 1: Figure S1(a) and Figure S1(b), the AR effect was much more pronounced in the double AR sample, as a result of the improvement of both abrupt interfaces of glass by the nanospheres. Click here for file

Additional file 2 **Isotherm of fresh and ageing suspension.** Ageing suspension gave a higher collapse pressure than fresh suspension with the same surfactant concentration. Click here for file

Additional file 3 **Compression-relaxation cycles.** The curve demonstrated that the monolayer of sphere on water is more compact after performing several compression-relaxation cycles. Click here for file

Additional file 4 **Influence of other parameters.** Influence of parameters including compression-relaxation cycles, dipper speed and annealing effect. Click here for file
