# Supplementary figures and images for: Tuning the peak position of subwavelength silica nanosphere broadband antireflection coatings
Source: Nanoscale Res Lett. 2014 Jul 19;9(1):361. doi: 10.1186/1556-276X-9-361 (PMC4128290; doi:10.1186/1556-276X-9-361)

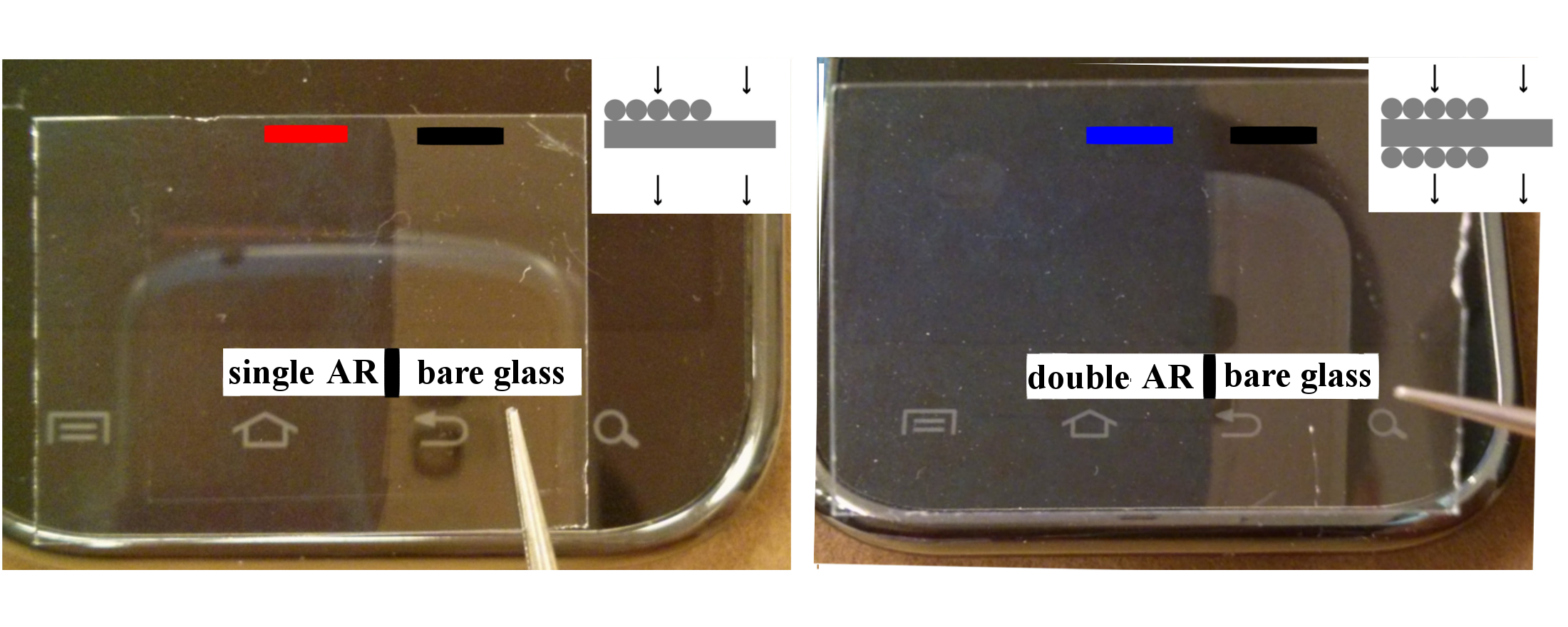

Supplement: Additional file 1 — Digital photographs of reflected images. In this figure, a mobile phone, which laid at the bottom, was used as the dark background. Glass samples with monolayer silica nanosphere coatings were laid on top of the mobile phone. A second smartphone with its built-in camera was used to take the photos. Therefore, the bare glass with high reflection would show the image of the photo-shooting smartphone camera. In Additional file 1: Figure S1(a), the left part of the glass sample was coated with single-side nanospheres, whereas in Additional file 1: Figure S1(b), both sides of the left part of the glass samples were coated with nanospheres. The right part of the glass in both Additional file 1: Figure S1(a) and S1(b) were left untreated for comparison, where reflecting image of the smartphone camera were clearly observed. The figure shows partially coated glass slides placed over mobile phone. Additional file 1: Figure S1(a) shows a glass slide with a silica nanosphere AR coating on a single side (single AR), while the glass slide on Additional file 1: Figure S1(b) is coated on both sides (double AR). Both samples are partially covered with the remaining glass left bare to observe the difference. These figures visually demonstrate two striking effects. Firstly, the transmittance of the coated glass is higher than the bare glass and is highest when the glass is coated on both sides (double AR). Secondly, the reflectivity, observed in the pictures as the reflection of the photo-taking camera, is reduced on the coated samples. No reflected image could be found on the double AR part of glass region in Additional file 1: Figure S1(b). Comparing Additional file 1: Figure S1(a) and Figure S1(b), the AR effect was much more pronounced in the double AR sample, as a result of the improvement of both abrupt interfaces of glass by the nanospheres. [file 1556-276X-9-361-S1.tif]

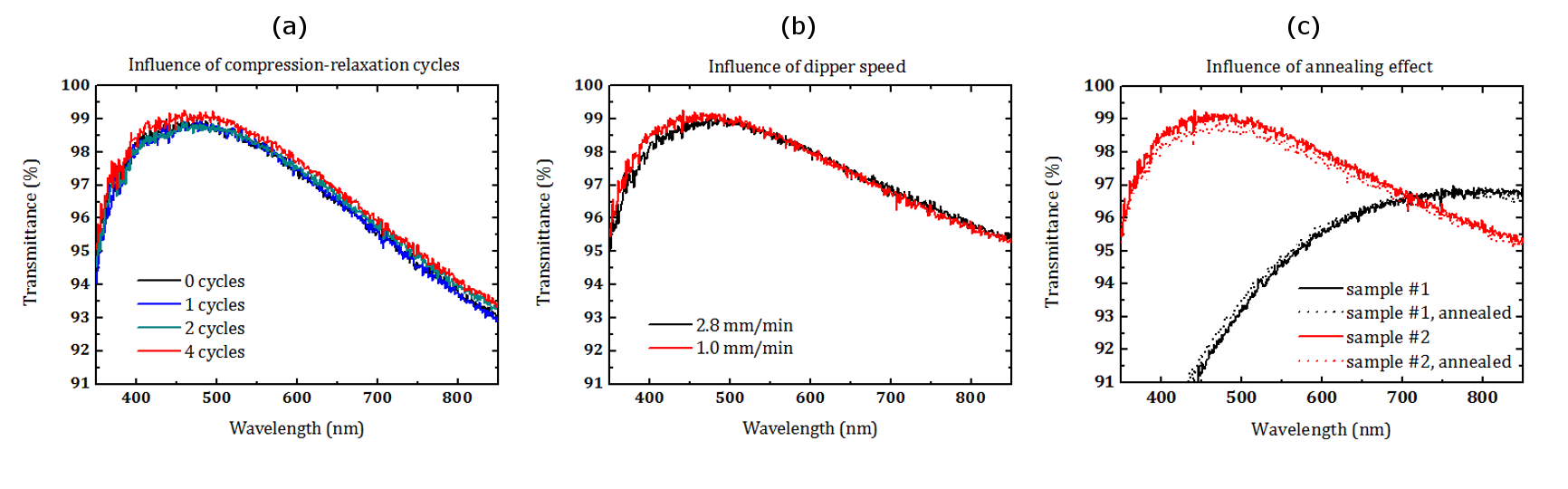

Supplement: Additional file 4 — Influence of other parameters. Influence of parameters including compression-relaxation cycles, dipper speed and annealing effect. [file 1556-276X-9-361-S4.tiff]
